# Artificial Intelligence–guided Extra-pulmonary Vein Repeat Ablation with the Pentaspline Pulsed Field Ablation Catheter

**DOI:** 10.19102/icrm.2026.17045

**Published:** 2026-04-15

**Authors:** Devi G. Nair, Kirollos Gabrah, Ganesh Nair, Brandon Doty, Arun Mahtani

**Affiliations:** 1 St. Bernards Medical Center and Arrhythmia Research Group, Jonesboro, AR, USA; 2 Arrhythmia Research Group, Jonesboro, AR, USA

**Keywords:** Artificial intelligence, catheter ablation, persistent atrial fibrillation, pulsed field ablation, spatiotemporal dispersion

## Abstract

The safety and efficacy of pulsed field ablation (PFA) for cardiac ablation have been demonstrated in pulmonary vein (PV) isolation (PVI), but its relevance to target additional extra-PV substrate is still under investigation. We conducted a pilot, prospective, single-center, nonrandomized study in 25 patients with atrial fibrillation despite previous catheter ablation (52% men; 74 ± 9 years of age). Patients underwent catheter ablation using PFA with the goal of ablating artificial intelligence (AI)-guided spatiotemporal dispersion. Biatrial mapping of spatiotemporal dispersion was obtained using the Volta AF-Xplorer™ (Volta Medical, Providence, RI, USA). Re-conducting PVs and extra-PV regions of interest exhibiting dispersion were ablated using the Farawave™ catheter (Boston Scientific, Marlborough, MA, USA). This catheter was used either in the basket or flower configuration sequentially centered at an estimated geometric center of AI-detected dispersion regions. The mean procedure and biatrial mapping times were 75 ± 12 and 14 ± 7 min, respectively, and no fluoroscopy was used. No complications occurred, and sinus rhythm conversion by ablation was obtained in 23 patients (92%). The rate of freedom from any atrial arrhythmia at 6 months was 88%. In conclusion, we observed that PFA for personalized, AI-guided extra-PV dispersion repeat ablation appears safe and procedurally efficient.

## Introduction

While atrial fibrillation (AF) is the most common arrhythmia in adults, its management remains suboptimal, and the most suitable catheter ablation strategy for re-ablation in AF patients is still elusive. While radiofrequency (RF) and cryoballoon thermal energies have been associated with debilitating complications,^1,2^ relatively nonthermal pulsed field ablation (PFA) has proven safe for pulmonary vein (PV) isolation (PVI).^3^ However, the safety and efficacy of using PFA to target extra-PV regions are still under investigation.^4–6^ The TAILORED-AF (“Tailored vs. Anatomical Ablation Strategy for Persistent Atrial Fibrillation”) trial, a large international randomized controlled trial, demonstrated for the first time that an electrogram (EGM)-based artificial intelligence (AI)-guided ablation strategy in addition to PVI is superior to PVI alone. Still, in this trial, ablation was conducted with RF energy and associated with a large increase in procedure time versus PVI alone.^7^ Here, we hypothesized that adopting a tailored workflow, consisting of PVI plus AI-guided dispersion ablation using PFA, is safe and efficient.

## Materials and methods

**Figure 1:**
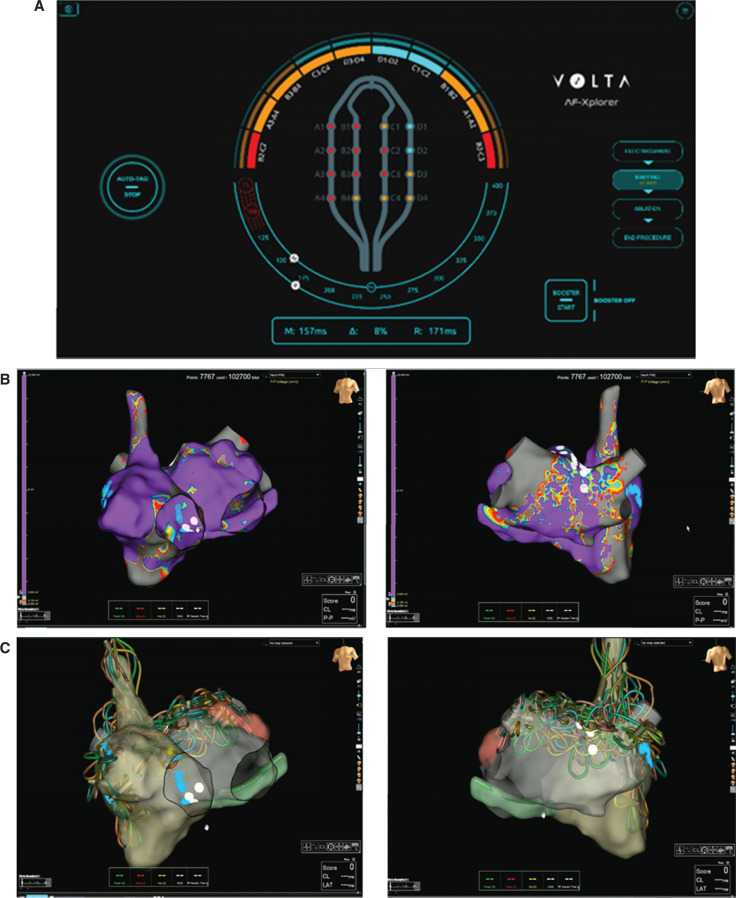
Artificial intelligence–guided pulsed field ablation workflow for repeat catheter ablation procedure. **A:** Representative picture of the Volta AF-Xplorer™ interface with the Advisor™ HD Grid mapping catheter. Red and orange signals correspond to a very high likelihood and high likelihood of dispersion, respectively. Dispersion tags are collected by placing the catheter in a position for a few seconds, and tags are added on the three-dimensional shell of the atrium in the mapping system. **B:** Representative pre-ablation dispersion map with anteroposterior and posteroanterior views. **C:** Representative post-ablation dispersion map with anteroposterior and posteroanterior views.

We performed a prospective, single-center, nonrandomized pilot study. This study was conducted in accordance with the principles of the Declaration of Helsinki. Persistent AF patients with recurrent persistent AF despite previous catheter ablation and maximum tolerated anti-arrhythmic drug (AAD) therapy were enrolled after providing oral consent from April 2024 to January 2025. During the index procedure, all patients (n = 25) had PVI, and eight patients (32%) underwent additional ablation, such as linear or EGM-based ablation. Electroanatomic mapping was performed with either the CARTO® mapping system (J&J MedTech, New Brunswick, NJ, USA) and the Octaray™ multipolar catheter (Biosense Webster, Diamond Bar, CA, USA) or EnSite™ X and the Advisor™ HD Grid catheter (both Abbott, Chicago, IL, USA). The expertise-based AI software Volta AF-Xplorer™ (Volta Medical, Providence, RI, USA) was used to detect spatiotemporal dispersion in multipolar intracardiac EGMs **(Figures 1A and 1B)**.^8^ The Volta AF-Xplorer™ algorithm integrates a knowledge-based classification model (extreme gradient boosting algorithm) and a deep-learning classification algorithm (convolutional neural network). Trained on >275,000 annotated EGMs, the system performs real-time signal analysis and provides visual and audio cues to assist operators. Algorithmic performance was evaluated using test datasets annotated by expert electrophysiologists.^7^ Spatiotemporal dispersion is defined as an ensemble of intracardiac EGMs forming a localized sequential activation in a distinct area, in which clusters of three or more adjacent bipolar EGMs show intracardiac activation spanning the entire AF cycle length. After biatrial mapping, PV conduction was evaluated, and PVs were re-isolated when relevant. Then, spatiotemporal dispersion ablation guided by Volta AF-Xplorer™ was performed using the Farawave™ catheter (Boston Scientific, Marlborough, MA, USA). The Farawave™ catheter was iteratively centered on the dispersion regions either in the basket or flower configuration. While ensuring optimal tissue contact with intracardiac echocardiography,^9^ the catheter was centered at the approximate geometric center of dispersion regions. A cavotricuspid isthmus line was performed in case of a documented history of common flutter or if a subject developed a common flutter during the procedure. Intracardiac echocardiography was used for real-time visualization, guidance of catheter positioning, and ablation energy delivery. Nitroglycerin was administered to prevent coronary artery spasm during ablation near the annulus, and intravenous hydration up to 1 L was provided to maintain hemodynamic stability and support renal function throughout the procedure.

Patients underwent 14-day ambulatory monitoring at 3 and 6 months.

## Results

All 25 participants underwent a repeat procedure during this study following prior catheter ablation in accordance with study enrollment criteria. The average age among study participants was 74 ± 9 years and 12 (48%) patients were female, with an average body mass index of 32 ± 8 kg/m^2^. Twenty-two (88%) patients presented with hypertension, 14 (56%) presented with heart failure, and 10 (40%) presented with diabetes, and the average CHA_2_DS_2_-VASc score was 5 ± 2 points **(Table 1)**. Sixteen patients were in spontaneous AF (64%) and three (12%) were in atrial flutter at the outset of the procedure, while AF was induced in six patients (24%). The mean procedure and biatrial mapping times were 75 ± 12 and 14 ± 7 min, respectively. No fluoroscopy was used, and no complications occurred. PVI was achieved and spatiotemporal dispersion was targeted for ablation in all 25 patients, with a mean of 56 ± 4 applications. Reconnection of PVs was observed in only one patient, with termination achieved at one of the four identified dispersion sites. Furthermore, 84% of patients had dispersion in the left atrium and 28% had such in the right atrium—specifically, 72% of the patients had dispersion in the anterior wall, 76% had it in the posterior wall, and 56% had it in the roof. Cavotricuspid isthmus and mitral lines were performed in 20% of patients. Sinus rhythm conversion by ablation was achieved in 23 patients (92%), of whom six patients (24%) first organized to flutter and two patients (8%) were cardioverted, and AF was finally non-inducible in all patients. AF was terminated at dispersion sites for all patients.

**Table 1:** Patient Baseline Characteristics

Baseline Characteristics	n = 25
Age (years)	74 ± 9
Sex, female, n (%)	12 (48%)
BMI (kg/m^2^)	32 ± 8
Hypertension, n (%)	22 (88%)
Heart failure, n (%)	14 (56%)
Diabetes mellitus, n (%)	10 (40%)
CHA_2_DS_2_-VASc score (points)	5 ± 2
Persistent, n (%)	25 (100%)

*Abbreviation:* BMI, body mass index. Data are expressed as mean ± standard deviation values or as n (%).

After a follow-up of 6 months (184 ± 10 days), the rate of freedom from any atrial arrhythmia was 88%. In total, three patients relapsed: one in AF and two in atrial tachycardia (AT). The case of AF spontaneously converted after 12 min, while both AT episodes lasted <1 h and converted with AAD administration.

## Discussion

### Workflow efficiency

In this pilot study, we demonstrate for the first time that personalized redo ablation represents an efficient workflow when a large footprint pentaspline PFA catheter is implemented. Also, we show that adding a procedural step of biatrial spatiotemporal dispersion mapping, with either the Octaray™ or the Advisor™ HD Grid multipolar catheter **(Figure 1)**, is not mutually exclusive with an expedited procedure. The TAILORED-AF trial was the first large-scale trial to demonstrate the superiority of PVI plus AI-guided dispersion ablation over the standard PVI-only procedure. In this trial, however, procedure times were prolonged, mostly because of the necessary point-by-point RF ablation in extra-PV regions (procedure time, 178 ± 60 vs. 92 ± 36 min in the PVI-alone group; RF time, 42 ± 17 vs. 20 ± 11 min in the PVI-alone group).^7^ Here, when using a PFA pentaspline catheter, the total procedure time for a tailored workflow of PVI plus AI-guided dispersion ablation in a repeat ablation population was just over 1 h. Interestingly, the procedure times reported here are on par with the ones reported in the MANIFEST-PF registry (“Multi-National Survey on the Methods, Efficacy, and Safety on the Post-Approval Clinical Use of Pulsed Field Ablation”) (average time: 61 [15–362] min in paroxysmal and persistent AF patients undergoing PFA catheter ablation)^3^ while being much shorter than those reported in the REAL-AF registry (“Real-world Experience of Catheter Ablation for the Treatment of Symptomatic Paroxysmal and Persistent Atrial Fibrillation”) (95 ± 42 min in paroxysmal AF patients undergoing RF catheter ablation).^10^ Further, in the ADVANTAGE AF trial (“Prospective Single Arm Open Label Study of the FARAPULSE Pulsed Field Ablation System in Subjects with Persistent Atrial Fibrillation”), the total procedure times were longer than the ones found in the present study (103 ± 35 min), and the pentaspline catheter was used to perform a PVI plus posterior wall strategy in persistent AF patients.^6^ Finally, in the MANIFEST-REDO trial (“Multicenter Analysis of Pulsed Field Ablation in the Real World—REDO Procedures”), the procedure times averaged 107 ± 40 min, which included 20 ± 12 min of fluoroscopy. Overall, these results strongly support the contention that the pentaspline PFA catheter allows for extensive extra-PV ablation at a minimal efficiency cost.

### Long-term outcomes after pulsed field ablation–enabled dispersion ablation

This pilot study highlights that PFA-conducted ablation may be associated with favorable long-term outcomes after both index and repeat procedures. These results, however, are preliminary in nature and will require further investigations. Previously, Farnir et al. reported in a prospective single-center study that a tailored substrate-based PFA strategy led to 64% freedom from arrhythmias at 6 months (115 and 7 min for the procedural and fluoroscopy times, respectively), with no complications.^4^ For comparison, the real-world MANIFEST-REDO study investigated patients who underwent repeat ablation after AF or AT recurrence after initial PFA ablation, with an arrhythmia-free survival rate of 65% after 284 (90–366) days of follow-up and a 3% procedural complication rate.^11^ Finally, Maurhofer et al. assessed the outcomes of PFA repeat ablation in patients with persistent AF after unsuccessful thermal ablation. They reported a rate of freedom from arrhythmia recurrence of 50% after 12 months with a procedural duration of 94 (73.5–135.2) min.^12^

## Conclusions

In this pilot investigation, we observed that extra-PVI spatiotemporal dispersion pulsed field repeat ablation appears safe and efficient. Further studies are needed to confirm whether a personalized PFA ablation approach may be associated with shorter procedure times, high safety standards, and superior long-term outcomes.
